# Comprehensive knowledge about cervical cancer is low among women in Northwest Ethiopia

**DOI:** 10.1186/1471-2407-13-2

**Published:** 2013-01-02

**Authors:** Frehiwot Getahun, Fekadu Mazengia, Mulunesh Abuhay, Zelalem Birhanu

**Affiliations:** 1 Department of Nursing, College of Medicine and Health Sciences, University of Gondar, Gondar, Ethiopia; 2 Department of Midwifery, College of Medicine and Health Sciences, University of Gondar, Gondar, Ethiopia; 3 Department of Reproductive Health, Institute of Public health, University of Gondar, Gondar, Ethiopia

**Keywords:** Cervical cancer, Women, Knowledge

## Abstract

**Background:**

Cervical cancer is the first most common cancer in women in sub-Saharan Africa followed by breast cancer. In Ethiopia, the incidence of cervical cancer is high i.e. 35.9 per 100,000 women. Low level of awareness, lack of effective screening programs, overshadowed by other health priorities (such as acquired immune deficiency syndrome, tuberculosis and malaria) and insufficient attention to women’s health are the possible factors for the observed higher incidence rate of cervical cancers in the country. Data on knowledge of Ethiopian women regarding cervical cancer is lacking. The aim of this study was to assess the knowledge of women about cervical cancer and associated factors.

**Methods:**

A community based cross-sectional survey was conducted from April 4-16, 2010 in Gondar town, Northwest Ethiopia. A total of 633 women aged 15 years and above were interviewed using semi-structured questionnaire by 8 trained data collectors and 2 supervisors. SPSS Windows version 15.0 was employed for data entry and analysis.

**Result:**

Of all the respondents, 495 (78.7%) of them had heard about cervical cancer and only 195 (31%) of them were knowledgeable about the disease.

**Conclusion:**

The knowledge of women on cervical cancer was found to be poor. Education about the disease must include information on risk factors, sign and symptoms of cervical cancer.

## Background

Cancers that originate in the female reproductive system are called women’s reproductive cancers. These include cancer of the cervix, breast, ovaries, vagina, vulva and endometrium [1]. Breast and cervical cancer are the most frequently occurring type of reproductive cancers in women worldwide [2]. Cervical cancer, a complication of Human Papillomavirus (HPV) infection, is the second most common cancer in women with 529,000 new cases each year worldwide. Eighty percent of the cases occur in low-resource countries like Africa, Latin America and Southeast Asia [3]. It is also a leading cause of mortality worldwide with 270 000 women every year. But, 85% of these deaths occur in the developing world [4].

According to the 2009 World Health Organization (WHO) report, the age-adjusted incidence rate of cervical cancer in Ethiopia is 35.9 per 100,000 patients with 7619 annual number of new cases and 6081 deaths every year [5]. Despite this fact, very few women receive screening services in Ethiopia [6]. Although there is no national cancer registry, reports from retrospective review of biopsy results have shown that cervical cancer is the most prevalent cancer among women in the country followed by breast cancer [7]. Low level of awareness, lack of effective screening programs, overshadowed by other health priorities (such as AIDS, TB, malaria) and insufficient attention to women’s health are the possible factors for the observed higher incidence rate of cervical cancer in the country [8] .

One major determinant for the prognosis of cervical cancer is the stage at which the patient presents [9]. Most patients in developing countries including Ethiopia present late with advanced stage disease, in which treatment may often involve multiple modalities including surgery, radiotherapy, chemotherapy, and has a markedly diminished chance of success [2]. Several factors such as educational status, financial capability, location, presence of health care facilities determine the stage at which patients with cancer present to the health facility. However, a common denominator of these factors is the level of awareness and attitude patients have about the diseases [10]. There is an increased chance of presenting early for treatment if patients have awareness about the disease [9].

Data on knowledge of Ethiopian women regarding cervical cancer is lacking. The lack of previous assessments limits the development and effectiveness of cancer prevention efforts. Provision of baseline information about the level of knowledge about cervical cancer among women in the community can assist program planners and health educators to target and tailor prevention programs.

## Methods

A community based cross-sectional survey was conducted among women of age 15 and above in Gondar town from April 16-26, 2010. Gondar town is found in North Gondar Zone of Amhara Regional State and is located 750 km Northwest of Addis Ababa. According to the 2007 Ethiopian census report, Gondar has a total population of 206, 987 and more than half (108,902) of them were females [11]. The town is divided into 12 administrative areas. There are two hospitals (1 referral and 1 defense hospital), 5 health centers and 1 NGO clinic.

A single population proportion formula, [n = (Z a/2)2 p (1-p)/d2], was used to estimate the sample size of women to be interviewed. The following assumptions have been made: proportion of women having knowledge about cervical cancer as 26.8% (p = 0.26), design effect of 2, 95% confidence interval, and margin of error to be 5% (d = 0.05) and 5% non response rate. Computing with the above formula gives a total sample size of 633.

Multi-stage sampling technique was utilized. Gondar Town has a total of 12 administrative areas. Four administrative areas were selected randomly. The number of households to be included in each administrative area was determined in proportion with the total number of households found in each administrative area. Then, a systematic random sampling method was employed to select the households. Whenever more than one eligible respondent (women of >15 years of age who were residing in the randomly selected administrative areas of Gondar town) was found in the same selected household, only one respondent was chosen using the lottery method. In case no eligible candidate was identified in a selected household or the selected household was closed even after three visits, the interviewer went to the next household in the clockwise direction until getting an eligible woman.

A structured questionnaire prepared in Amharic was used for this study. It was adopted from a survey tool developed by the American cancer society through some modifications. Before the actual data collection, pretest was conducted with 31 households in Azezo administrative area and necessary corrections were made. Eight trained female nurses collected data via interview. The principal investigator and two public health officers supervised the data collection process. They made frequent checks on the data collection process to ensure the completeness and consistency of the gathered information.

The data were entered and analyzed using SPSS version 15 statistical package. Data cleaning was performed to check for accuracy, consistency and missed values. Frequencies, proportions and summary statistics were used to describe the study population in relation to relevant variables. The impact of selected socio demographic and other characteristics on knowledge of cervical cancer was investigated using both the bivariate method and the multivariate logistic regression technique. Finally, explanatory variables with p value of less than 0.2 in the bivariate analysis were included in the multiple logistic regressions. Odds ratio and 95% confidence interval were also used to identify the presence and strength of association.

Ethical clearance for the proposed study was obtained from the ethical review committee of College of Medicine and Health Sciences, University of Gondar. Communication with the different Town and kebele administrators was made through formal letter obtained from University of Gondar. The purpose and importance of the study was explained to the participants. Data were collected after full informed verbal consent was obtained. Confidentiality of the information was maintained throughout by excluding names as identification in the questionnaire and keeping their privacy during the interview by interviewing them alone.

## Result

### Socio demographic characteristics of the study population

A total of 633 women aged 15 and above years were included in the study making the response rate 100%. Six hundred twenty nine (99.4%) of them were eligible for the final analysis. Of the participants, 436(69.3%) were aged between 20-39 years with a mean age of 31 years (SD+11.3) and 281 (44.6%) were married. Nearly half of the respondents (53.6%) gave birth two or more times with mean parity of 2 (SD+2.2). Around 57.0% of the respondents attended at least high school. Orthodox was the dominant religion among the study participants (82.0%) (Table 1).

**Table 1:** Percentage distribution of the study population by selected socio demographic characteristics Gondar town, Ethiopia, April 2010

**No.**	**Variable**	**Frequency (N=629)**	**Percentage (100%)**	**Mean ±SD**
1	**Age**			Mean age ±SD 31.2±(11.3)
	15-19	57	9.1	
	20-24	159	25.3	
	25-29	126	20.0	
	30-34	73	11.6	
	35-39	78	12.4	
	40-44	49	7.8	
	45+	87	13.8	
2	**Marital status**			
	Single	227	36.1	
	Married	281	44.6	
	Divorced	52	8.3	
	Widowed	69	11.0	
3	**Religion**			
	Orthodox	516	82.0	
	Muslim	83	13.2	
	Catholic	7	1.1	
	Protestant	20	3.2	
	Others	3	0.5	
4	**Educational status**			
	Illiterate	118	18.8	
	Read & write	59	9.4	
	Primary education	94	14.9	
	High school	270	42.9	
	Diploma	73	11.6	
	Degree & above	15	2.4	
5	**Husband's education**	**N=260**	5.4	
	Illiterate	14	14.2	
	Read & write	37	15.4	
	Primary education	40	37.3	
	High school	97	17.3	
	Diploma	45	10.4	
	Degree & above	27		
6	**Occupation**			
	Government employee	120	19.1	
	NGO employee	15	2.4	
	Self employee/ merchant	164	26.1	
	Student	119	18.9	
	House wife	182	28.9	
	Retired	29	4.6	
7	**Parity**			Mean no. of children ±SD 2 ±(2)
	0	231	36.7	
	1	61	9.7	
	2-4	244	38.8	
	5+	93	14.8	
8	**Monthly household income**			Mean income ±SD 800±(805.00)
	Low	157	25.0	
	Medium	317	50.4	
	High	155	24.6	

### Knowledge of women on cervical cancer

About 495 (78.7%) of the respondents had heard about cervical cancer. When they were asked about the source of information, television/radio was the predominant source 301 (60.8%) followed by health professionals 173(34.9%) and friends/relatives 107 (21.6%). One hundred forty eight (23.5%) of the respondents also knew someone who has cervical cancer and 529 (84.1%) of the respondents have visited health institution for some reason.

**Figure 1:**
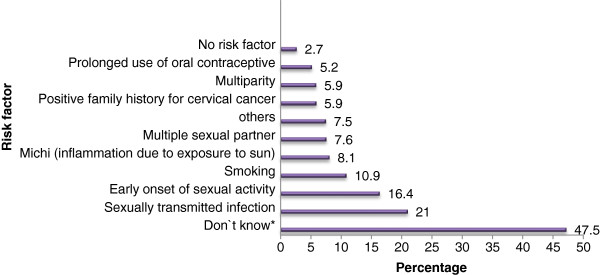
Respondents’ knowledge of risk factors for cervical cancer, April 2010, Gondar, Ethiopia.

A series of questions regarding risk factors, main symptoms, treatment options and prevention and early detection measures of cervical cancer were asked to evaluate the respondents’ knowledge about cervical cancer. About 47.5% of the respondents did not know whether there are risk factors for cervical cancer or not and 17 (2.7%) stated that there is no risk factor for cervical cancer. One hundred eighteen (18.8%) of the study participants were unable to mention a risk factor although they said that cervical cancer has a risk factor. In general, 195 (31.0%) of them were able to identify at least one risk factor for cervical cancer. STI and early onset of sexual activity were specific risk factors mentioned by 132(21.0%) and 103 (16.4%) of the respondents respectively (Figure 1).

Among all the participants, 222 (35.3%) and 187 (29.7%) of them mentioned offensive and excessive vaginal discharge respectively when asked about the symptoms of cervical cancer. However, 249 (39.6%) of the respondents did not know any symptom. Four hundred two (63.9%) of the respondents knew that cervical cancer can be prevented. Regular medical checkup (screening) was mentioned by 345 (54.8%) of the respondents as a helpful prevention measure. Four hundred sixteen (66.1%) of the respondents also knew that cervical cancer can be treated and 332 (52.8%) agreed that cervical cancer can be cured if detected early (Table 2).

**Table 2:** Knowledge of women about main presenting symptoms, Prevention measures and treatment options of cervical cancer, Gondar town, Ethiopia, April 2010

Symptoms*	**Number**	**Percent**
Bleeding and pain after sexual intercourse	99	15.7
Post menopausal bleeding	80	12.7
Excessive vaginal discharge	187	29.7
Offensive vaginal discharge	222	35.3
Abnormal bleeding between periods	91	14.5
Others	17	2.7
Don’t know	249	39.6
Prevention measures*	**Number**	**Percent**
Regular medical checkup/screening**	345	54.8
Vaccine for HPV	38	6.0
Delaying sexual debut	39	6.2
Being faithful to sexual partner	38	6.0
Consistent condom use	14	2.2
Others	23	3.7
Don’t know	227	36.1
Treatment options*		
Radiation therapy	171	27.2
Surgery	210	33.4
Chemotherapy	218	34.6
Others	3	0.5
Don’t know	213	33.9%
**Stage at which cervical cancer can be cured**		
Early	332	52.8
Cannot be cured at any time	94	14.9
Any time	6	1.0
Late	13	2.1
Do not know	184	29.1

402(63.9%) of the respondents knew that cervical cancer can be prevented.

*Percent may exceed 100% as multiple answers are possible.

**Data, that confirms whether women who had heard about Pap smear are included in the group that identified "regular medical checkup/screening" as one of the preventive measures, is not reported.

Questions regarding knowledge of risk factors, symptoms, treatment options and prevention and early detection measures for cervical cancer were scored and pulled together and the mean score was computed to determine the overall knowledge of respondents. Respondents scored average and above were considered as knowledgeable otherwise not. Only 195 (31.0%) of the total respondents were found to have above-average knowledge when the comprehensive cervical cancer knowledge was determined.

A significant difference on the knowledge of respondents among different groups of age, educational status, occupation, monthly household income, parity, knowing someone with cervical cancer and visit to a health institution was detected during the bivariate logistics regression analysis. But only secondary and above educational status, knowing someone with cervical cancer and visit to a health institution were shown to be significant predictors of knowledge when adjusted for variables p value less than 0.2. Participants with secondary and above education were also about 1.2 times more likely to be knowledgeable than women with no formal education [AOR=2.18, 95%CI (1.20-3.95). In addition knowing someone with cervical cancer [AOR=4.91, 95%CI (3.16-7.62)] and ever visit to health institution [AOR=8.13, 95%CI (3.19-20.75)] were also factors that are more likely to increase knowledge of cervical cancer (Table 3).

**Table 3:** Socio-demographic correlates of cervical cancer knowledge of women in Gondar town, Ethiopia, April 2010

**Variables**	**Knowledge of cervical cancer**		**Crude OR (95% CI)**	**Adjusted OR (95% CI)**
	**Yes**	**No**		
**Age**				
15-24	69(31.9%)	147(68.1%)	1.80(1.00-3.25)	0.71(0.45-1.71)
25-34	69(34.7%)	130(65.3%)	2.04(1.12-3.69)	1.09(0.30-1.72)
35-44	39(30.7%)	88(69.3%)	1.70(0.90-3.23)	0.85(0.39-1.87)
45+	18(20.7%)	69(79.3%)	1.00	1.00
**Marital status**				
Single	71(31.3%)	156(68.7%)	1.00	**
Married	91(32.4%)	190(67.6%)	1.05(0.72-1.53)	
Divorced	18(34.6%)	34(65.4%)	1.16(0.62-2.20)	
Widowed	15(21.7%)	54(78.3%)	0.61(0.32-1.15)	
**Educational status**				
No formal education	31(17.5%)	146(82.5%)	1.00	1.00
Primary education (1-8)	19(20.2%)	75(79.8%)	1.19(0.63-2.25)	0.96(0.47-1.96)
Secondary education & Above	145(40.5%)	213(59.5%)	3.21(2.06-4.99)	2.18(1.20-3.95)*
**Occupation**				
Employed	109(36.5%)	190(63.5%)	2.31(1.53-3.49)	1.55(0.94-2.54)
Student	44(37.0%)	75(63.0%)	2.36(1.43-3.90)	1.91(0.94-3.88)
Not employed	42(19.9%)	169(80.1%)	1.00	1.00
**Monthly household income**				
Low	40(25.5%)	117(74.5%)	1.00	1.00
Medium	78(24.6%)	239(75.4%)	0.96(.61-1.48)	0.77(0.47-1.29)
High	77(49.7%)	78(50.3%)	2.89(1.79-4.66)	1.53(0.85-2.74)
**Parity**				
0	79(34.2%)	152(6.8%)5	1.00	1.00
1	20(32.8%)	41(67.2%)	0.94(0.56-1.71)	0.98(0.46-2.10)
2-4	77(31.6%)	167(68.4%)	0.89(0.61-1.30)	0.88(0.50-1.56)
5+	19(20.4%)	74(79.6%)	0.49 (0.28-.88)	0.59(0.25-1.38)
Know anyone with cervical cancer				
Yes	88(59.5%)	60(40.5%)	5.13(3.46-7.59)	4.91(3.16-7.62)*
No	107(22.2%)	374(77.8%)	1.00	1
Ever visit to health institution				
Yes	190(35.9%)	339(64.1%)	10.65(4.26-26.63)	8.13(3.19-20.75)*
No	5(5.0%)	95(95.0%)	1	1

Average knowledge score=4.5.

*Statistically significant.

**Variables with p value of greater than 0.2 in crude analysis omitted from entering in to the multivariate model.

## Discussion

Knowing the causative/risk factors, symptoms and preventive measures of a particular cancer can make all the differences, without this prevention is far more difficult. Though there is lack of studies conducted in Ethiopia, studies conducted worldwide have shown that there is poor knowledge of both cervical and breast cancer among women [12]. In this study, 78.7% of women had heard about cervical cancer which is consistent with the study conducted in Ghana, Accra (93.0%). But higher than the findings from Nigeria (40.8%) [13,14]. This gap might be due to the difference in time period and nature of the population the two studies conducted, as the Nigeria study is conducted on rural women. Television/Radio was mentioned as the main source of information (60.8%) for cervical cancer by the respondents concurring with the finding of the study in Nigeria (46.4%) [14].

Only 31.0% of the study participants were able to identify at least one risk factor for cervical cancer like STIs, early onset of sexual activity, multiple sexual partner and smoking. A study done in South Africa showed that 64.0% of the respondents gave one or more correct risk factors [15]. The difference might be attributed to the fact that South Africa has a national cervical cancer screening policy. But in Ethiopia, there is no organized reproductive organ cancer prevention, education, screening, or curative care program, nor is there is any national policy to address this issue.

Prevention and early detection are keys to the reduction of incidence and progression of many chronic diseases including cancer [16]. Around two third (63.9%) of the respondents knew that cervical cancer can be prevented. This is higher than the South African study which 57.0% of the respondents knew that cervical cancer can be prevented [15]. This difference can be explained by the difference in the background of the study participants and the time gap as better attention has been given to cancer these days. Fifteen percent of the respondents believe that cervical cancer cannot be cured. This can be an indication of the presence of misconception about the disease in this community and may hinder prevention efforts.

Though majority of the respondents had heard about cervical cancer, only one third (31.0%) of the respondents were found to have above-average knowledge. Different studies from Nigeria (23.4%) and Ghana (37.0%) [14,13] also showed that comprehensive knowledge about cervical cancer is low.

In many studies, different socio demographic variables have shown an influence on knowledge of cervical cancer. In this study, women with secondary and above level of education were about two times more likely to have above-average knowledge than women with no formal education. The importance of educational status on knowledge of cervical cancer has been mentioned in a study done in Cameroon [17].

In addition, knowing someone with cervical cancer and ever visit to health institution were significant predictors of knowledge. Women who knew someone affected with cervical cancer were about five times more likely to have above-average knowledge than women who did not. Similarly women who ever visited health institution for any reason were eight times more likely to have above-average knowledge about cervical cancer than women who didn’t. This indicates that women who visited health institutions have a higher chance of getting more comprehensive information from health professionals in the form of health education or counseling or from people who are affected by the disease.

Most invasive cancers of the cervix can be prevented if women have Pap tests regularly. The general recommendation for Pap smear is each woman in high-risk target demographic groups is screened once before any woman is screened a second time [18]. In this study only 13.7% of the women had heard about Pap smear and only 14.7% of them had the test. This finding is consistent with the study in Nigeria where 73.0% of the women were not aware of the test and only 5.2% had the test [14]. Another Nigerian study also showed that only 7.1% of the respondents had the test [19]. But, the finding is lower than the study in South Africa where 49.0% of the respondents ever heard of the test and 18.0% had the test [15]. The difference is not surprising because pap smear is widely available as a screening tool in South Africa and there is also national pap smear policy where as pap test is available only in some health institutions in Ethiopia.

## Conclusion

The results of this study revealed that knowledge about cervical cancer was poor though majority of the women had heard about the disease. Specifically, the knowledge of women on risk factors, signs and symptoms was poor. Education about the disease must include information on risk factors, sign and symptoms of cervical cancer.

## Competing interests

The authors declare that they have no competing interests.

## Authors’ contributions

FG wrote the proposal, participated in data collection, analyzed the data and drafted the paper. FM, MA and ZB approved the proposal with some revisions, participated in data analysis and revised subsequent drafts of the paper. All authors read and approved the final manuscript.

## Pre-publication history

The pre-publication history for this paper can be accessed here:

http://www.biomedcentral.com/1471-2407/13/2/prepub
